# Expression of Concern: Cajanonic acid A regulates the ratio of Th17/Treg via inhibition of expression of IL-6 and TGF-β in insulin-resistant HepG2 cells

**DOI:** 10.1042/BSR-20181716_EOC

**Published:** 2021-02-17

**Keywords:** Cajanonic acid A, IL-6, insulin resistance, TGF-β, Th17/Treg

The Editorial Office has been made aware of potential issues surrounding the scientific validity of this paper, hence has issued an expression of concern to notify readers whilst the Editorial Office investigates.

